# An Unusual Cause of a Pancreatic Mass: Pancreatic Tuberculosis

**DOI:** 10.7759/cureus.4732

**Published:** 2019-05-23

**Authors:** Abdul Rehman, Abdul Majeed Maliyakkal, Khalifa L Farfar, Nabeel M Shaath, Vamanjore A Naushad

**Affiliations:** 1 Internal Medicine, Rutgers New Jersey Medical School, Newark, USA; 2 Internal Medicine, Hamad Medical Corporation, Doha, QAT; 3 Internal Medicine - Gastroenterology, Hamad Medical Corporation, Doha, QAT; 4 Emergency Medicine, Hamad Medical Corporation, Doha, QAT

**Keywords:** acid fast bacilli, endoscopic ultrasound, tuberculosis, mycobacterium tuberculosis, pancreatic tuberculosis

## Abstract

Isolated pancreatic tuberculosis (TB) is an exceedingly rare disease, even in countries with a high burden of TB. We report the case of a 40-year-old gentleman who presented with a two-week history of fever and abdominal pain. Computed tomography of the abdomen showed a large heterogeneous mass arising from the pancreas with peri-pancreatic lymphadenopathy, infiltration of the stomach, and encasement of the celiac vessels-highly suggestive of pancreatic malignancy. Oesophagogastroduodenoscopy with endoscopic ultrasonography was performed, which showed a necrotic area in the body of pancreas. Fine needle aspiration from the necrotic area was positive for *Mycobacterium tuberculosis* by polymerase chain reaction (PCR)* *and culture methods. This case demonstrates an unusual presentation of TB with predominant involvement of the pancreas in the absence of pulmonary or systemic involvement. Clinicians need to be aware of this unique presentation of tuberculosis in order to avoid misdiagnosis and institute timely treatment.

## Introduction

Tuberculosis (TB) is a chronic granulomatous infection caused by the fastidious organism *Mycobacterium tuberculosis *(*M. tuberculosis*). According to the Centers for Disease Control and Prevention (CDC), approximately 10.4 million people were affected by TB in the year 2015, accounting for 1.8 million deaths worldwide during 2015 [1]. The developing world bears a disproportionate burden of this disease as the risk factors for this disease, such as poverty, overcrowding and malnutrition, are common in lower-to-middle income countries. *M. tuberculosis* preferentially affects the lungs as it is an obligate aerobe and airborne transmission of this microbe occurs readily, which accounts for the highly contagious nature of TB. While extra-pulmonary manifestations of TB are well described, isolated pancreatic TB remains a rare entity [2]. Here, we report the case of a 40-year-old gentleman who presented with fever, abdominal pain and weight loss, and he was subsequently diagnosed with pancreatic TB.

## Case presentation

A 40-year-old Nepalese gentleman presented to the emergency department with complaints of fever, night sweats and abdominal pain for the past two weeks with associated anorexia and significant weight loss (lost 7 kg in one month). The patient denied any change in urinary or bowel habits, cough, sputum production, rash or shortness of breath. He did not report any features suggestive of steatorrhea, such as loose, greasy foul-smelling stools. His past medical and surgical history was unremarkable and he did not have any hospitalizations in the past. Family history was significant for pulmonary TB in his mother when the patient was five years old. He was a non-smoker and consumed alcohol occasionally.

On physical examination, he appeared underweight (BMI 18.8 kg/m^2^) and mildly dehydrated. His vital signs were: pulse rate of 120/min (regular), blood pressure 100/60 mm Hg, respiratory rate 20/min and an oral temperature of 39.1°C. He had mild pallor but did not have scleral icterus or lymphadenopathy. Abdominal examination revealed epigastric fullness with mild tenderness without any palpable mass. Examination of the pulmonary, cardiac and genitourinary systems was within normal limits.

**Figure 1:**
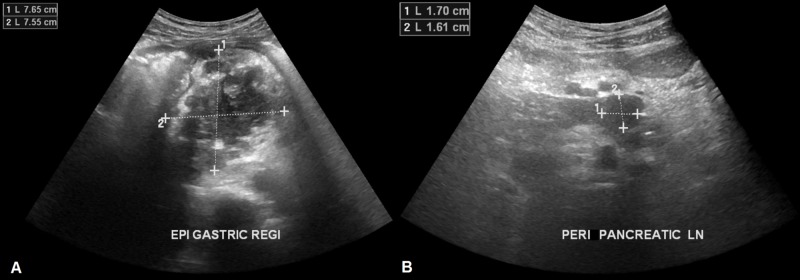
Images of abdominal ultrasonography (A) A large (7.6 cm × 7.7 cm) heterogeneous lesion is seen arising from the pancreas. (B) A peri-pancreatic lymph node is visible measuring 1.7 cm × 1.6 cm in size.

**Figure 2:**
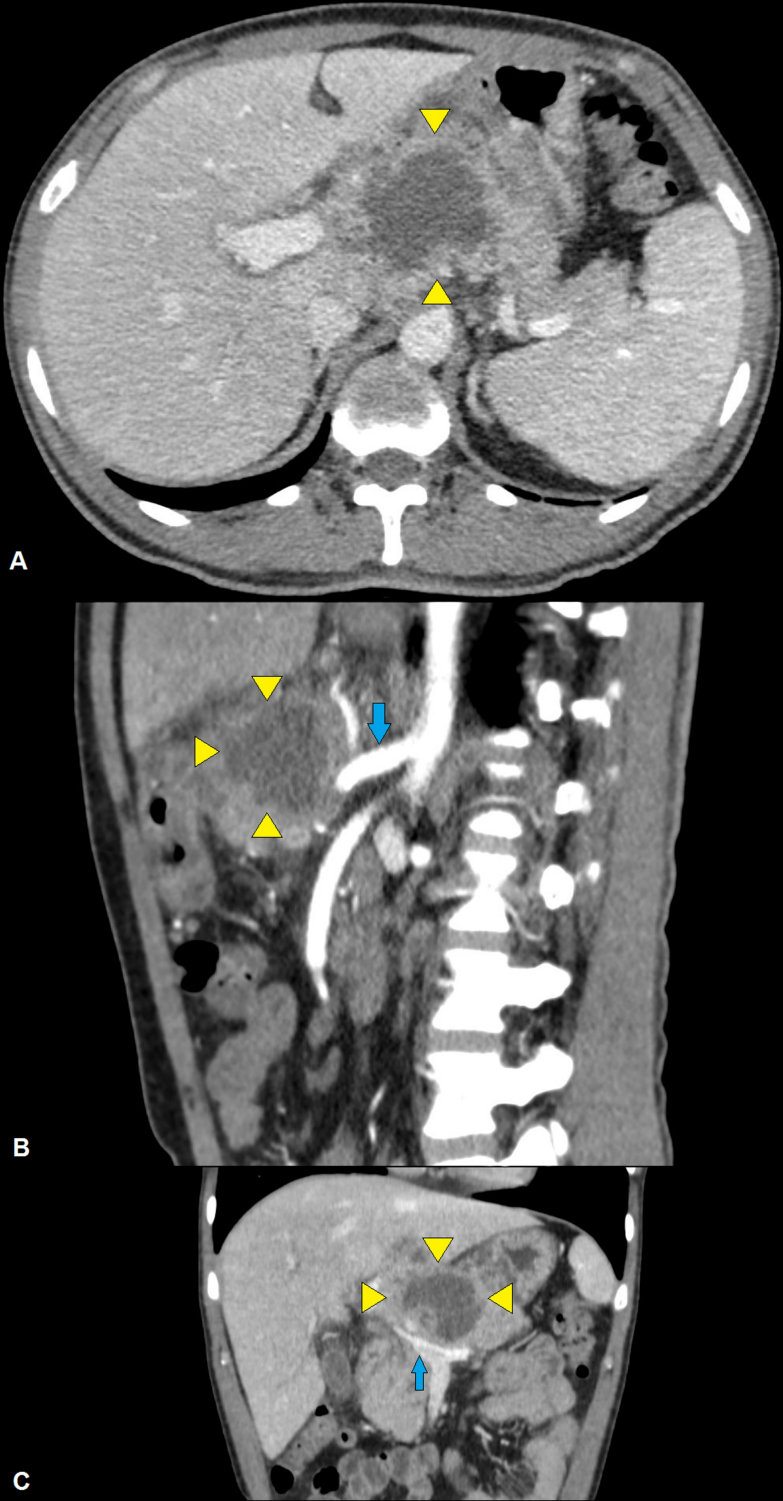
Computed tomography of the abdomen (A) A large heterogeneous necrotic mass (arrowheads) is seen arising from the pancreatic body with loss of fat planes and possible infiltration of the left lobe of the liver. (B) The mass (arrowheads) encases the celiac vessels (arrow) and (C) portal vein (arrow).

Patient’s laboratory investigations are given in Table 1. Chest radiograph and electrocardiogram were within normal limits. Two sets of blood cultures (drawn peripherally) showed no growth at five days. Tuberculin test and interferon-γ release assay (QuantiFERON-TB Gold™; Qiagen® Inc., Hilden, Germany) were negative. A chemiluminescent microparticle immunoassay (Architect® HIV Ag/Ab Combo; Abbott Diagnostics, Lake Forest, IL) for human immunodeficiency virus (HIV) p24 antigen and HIV-1 & HIV-2 antibodies was also negative. Abdominal ultrasonography (day 1) showed a large (7.6 cm × 7.7 cm), heterogeneous, predominantly solid lesion arising from the pancreas and encasing the celiac vessels, raising the suspicion of pancreatic malignancy (Figure 1). Computed tomography (CT) of the abdomen (with contrast) revealed a large (6 cm × 5.6 cm × 4.6 cm) heterogeneous necrotic mass in the lesser sac arising from the pancreatic body with loss of fat planes, a possible infiltration of the greater curvature of the stomach and left lobe of liver, and encasement of celiac vessels and portal vein (Figure 2). Multiple peri-pancreatic and retroperitoneal lymph nodes with necrotic centers and peripheral enhancements were also seen.

**Table 1:** Results of laboratory investigations at baseline * Abnormal results are marked with an asterisk (*)

Creatinine	72 μmol/L	64–110 μmol/L
Alanine aminotransferase	53 U/L	0–55 U/L
Aspartate aminotransferase*	59 U/L*	5–34 U/L
Alkaline phosphatase*	176 U/L*	40–150 U/L
Gamma-glutamyl transferase*	50 U/L*	10–29 U/L
Total bilirubin	15 μmol/L	3.4–20.5 μmol/L
Albumin*	24 g/L*	35–50 g/L
Lipase	22 U/L	8–78 U/L
C-reactive protein*	246 mg/L*	0–5 mg/L
Cortisol (morning)	478 nmol/L	138–635 nmol/L
25-hydroxy-cholecalciferol*	8 ng/mL*	30–80 ng/mL
Carbohydrate antigen (CA) 19-9	16 U/L	0–37 U/L

**Figure 3:**
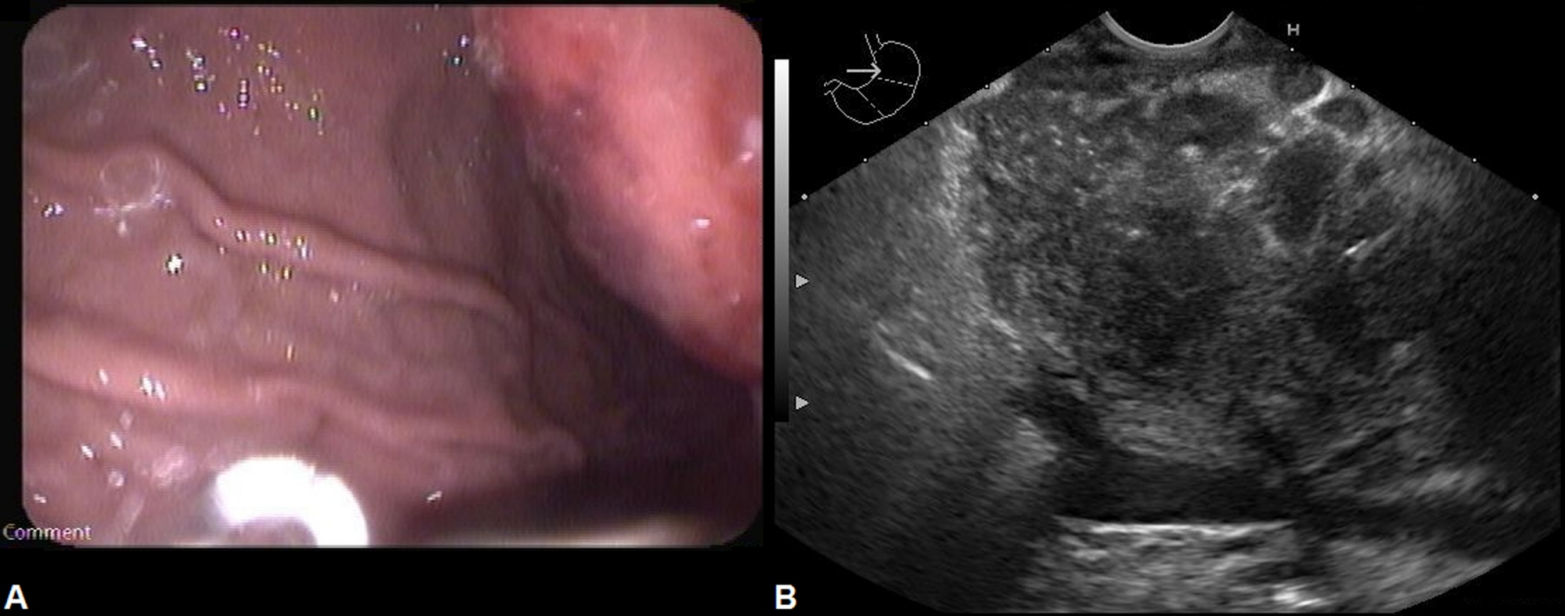
Gastroscopy with endoscopic ultrasound (A) Gastroscopy shows an extrinsic mass causing compression and visible indentation within the lumen of the stomach. (B) Image of endoscopic ultrasonography demonstrating an ill-defined necrotic mass within the pancreas.

Based on the results of radiologic studies, a primary pancreatic malignancy was considered the most likely possibility. However, the patient’s relatively short history of constitutional symptoms was inconsistent with this diagnosis and other differential diagnoses were also considered. These included infective causes (brucellosis, tuberculosis, actinomycosis and fungal infections), inflammatory disorders (auto-immune pancreatitis), lymphoma and systemic lupus erythematosus. The gastroenterology service was consulted and the decision was made to proceed with oesophagogastroduodenoscopy (OGD) with endoscopic ultrasonography (EUS). OGD with EUS revealed an ill-defined necrotic mass in the body of the pancreas (measuring 3 cm × 3 cm) which was compressing the stomach externally (Figure 3). Multiple peri-pancreatic lymph nodes were also seen. EUS-guided fine-needle aspiration was performed from the pancreatic mass and adjacent lymph nodes using a 25-gauge needle. Cytopathology revealed granulomatous inflammation with aggregates of epithelioid cells and some multinucleated giant cells. There was no evidence of malignancy or atypical cells. Acid fast bacilli (AFB) smears and gram-stain of aspirated fluid were negative. Qualitative, nested real-time polymerase chain reaction (GeneXpert® MTB/RIF Assay; Cepheid Inc., Sunnyvale, CA) of aspirated fluid was positive for *M. tuberculosis*. Mycobacterial culture (BACTEC™ MGIT 960™ system; Becton Dickinson [BD] Medical, Franklin Lakes, NJ) of the aspirated fluid also grew *M. tuberculosis*.

When the patient initially came to the emergency department, he was started on intravenous hydration, and empiric antibiotics (ceftriaxone and metronidazole) to cover for any intra-abdominal infection. He was subsequently admitted to the medical floor and continued to receive supportive treatment for the next four days without any improvement in his condition. On day five of admission, after confirmation of tuberculosis by polymerase chain reaction (PCR), his antibiotics were discontinued and he was started on first-line anti-tuberculous therapy (isoniazid 300 mg/day × 26 weeks, rifampin 600 mg/day × 26 weeks, pyrazinamide 1600 mg/day × 8 weeks and ethambutol 1100 mg × 8 weeks) along with pyridoxine (50 mg/day) and paracetamol. At the time of initiation of anti-tuberculous therapy, results of AFB culture were pending; later, the result of AFB culture provided further confirmation of the diagnosis. His condition gradually improved and he was discharged home with a plan of outpatient follow-up. At the time of discharge, the patient was started on oral cholecalciferol 50,000 units once weekly for eight weeks.

At a one-month follow-up visit, the patient’s clinical symptoms had improved markedly and he gained 2 kg weight. Repeat laboratory investigations showed improvement in his total leukocyte count and inflammatory markers (see Table 2). The patient completed anti-tuberculous therapy uneventfully and continued to lead a healthy life. At a 10-month follow-up visit, he was completely asymptomatic and his BMI had improved to 22.9 kg/m^2^ (from 18.8 kg/m^2^ at the time of his first visit).

**Table 2:** Results of laboratory investigations at the end of the treatment

Tests	Results	Reference range
Hematology		
Hemoglobin	13.2 g/dl	13–17 g/dl
White cell count	5.8 × 10^9^/L	4–10 × 10^9^/L
Platelet count	223 × 10^9^/L	150–400 × 10^9^/L
Erythrocyte sedimentation rate	11 mm/hr	2–28 mm/hr
Biochemistry		
Creatinine	68 μmol/L	64–110 μmol/L
Alanine aminotransferase	23 U/L	0–55 U/L
Aspartate aminotransferase	28 U/L	5–34 U/L
Alkaline phosphatase	122 U/L	40–150 U/L
Total bilirubin	18 μmol/L	3.4–20.5 μmol/L
Albumin	38 g/L	35–50/L
C-reactive protein	5 mg/L	0–5 mg/L
25-hydroxy-cholecalciferol	35 ng/mL	30–80 ng/mL

## Discussion

Pancreatic TB is a rare but well-described entity. Abdominal TB involving the peritoneum and intestines (particularly the ileocecal junction) has been described in numerous studies. However, the literature on pancreatic TB has been limited to a small number of case reports and case series [3]. It has been hypothesized that pancreatic enzymes (such as lipase and deoxyribonuclease) inhibit the growth of mycobacteria, which may account for the relatively low incidence of this disease (even in endemic areas). Nevertheless, cases of pancreatic TB have been reported more frequently in the past decade. This may be a consequence of both increased recognition of this distinct disease and an increase in the incidence of the disease due to a surge of cases of acquired immunodeficiency syndrome (AIDS). While pancreatic TB has been more frequently described in patients with AIDS, cases of pancreatic TB in healthy adults have also been reported [4]. Interestingly, our patient did not suffer from any immunosuppressive state such as AIDS. Moreover, a substantial proportion of cases of pancreatic TB have been described in relatively young patients (mean age of 40 years) [5]. Our patient was a healthy 40-year-old gentleman with no known comorbidities and no significant past medical history. The exact reasons for this observation remain unclear.

Diagnosis of pancreatic TB is challenging in most cases and often poses a dilemma for most treating physicians. Radiologic investigations are necessary to delineate the involvement of the pancreas and other intra-abdominal organs. CT may depict nodules within the pancreas more accurately and demonstrate the involvement of peri-pancreatic and/or retroperitoneal lymph nodes [6]. CT is also useful in that it may reveal the involvement of other abdominal viscera and possibly peritoneal enhancement. However, none of these radiologic features are sufficiently specific to distinguish between pancreatic TB and malignancy [7]. As in our case, radiologic appearance of pancreatic TB can masquerade as pancreatic malignancy, which may lead to confusion and possible misdiagnosis. In such cases, EUS has been advocated as the modality of choice for diagnosis as it enables adequate visualization of the pancreas and allows tissue sampling for microbiologic and histopathological confirmation [8]. CT-guided fine-needle aspiration (FNA) and/or biopsy are alternative methods to obtain specimens for microbiologic and histopathological evaluation, and has been utilized for diagnosis in certain cases [9]. The classic histopathological diagnostic features of TB are granulomatous inflammation with caseous necrosis and/or direct visualization of acid fast bacilli (AFB) in tissue specimens. In practice, the presence of granuloma is the most common feature and often diagnostically useful in the appropriate clinical context [10]. Microbiologic methods of diagnosis include staining for AFB using auramine-rhodamine stain, Kinyoun stain and/or Zeel-Nielsen stain, and direct culture of mycobacteria using solid (e.g. Lowenstein-Jensen medium) or liquid (e.g. Middlebrook 7H9 broth) media. In some cases, laparotomy with pancreatic biopsy was performed when both AFB stains and cultures were negative [11]. A newer cartridge-based nucleic acid amplification test (viz. GeneXpert® MTB/RIF Assay) was endorsed by the World Health Organization (WHO) in 2010 for diagnosis of pulmonary and extra-pulmonary TB [12]. This assay has also been approved by the United States Food and Drug Administration (FDA) for clinical practice, albeit only for use on sputum specimens [13]. In the present case, rapid diagnosis of pancreatic TB was made possible through the use of this assay (on aspirated pancreatic fluid), which allowed for prompt institution of anti-tuberculous therapy. Subsequent culture of *M. tuberculosis* from the aspirated fluid further validated this diagnosis. The present case (as well as few other cases reported previously [8, 11]) suggests that the use of molecular-based methods in the diagnosis of extra-pulmonary TB (including pancreatic TB) can be invaluable and may obviate the need for more invasive procedures (such as laparotomy).

The pathogenesis of TB has been extensively studied over the past few decades and many aspects have been well described, although a few questions remain unsettled. Most adult cases of pulmonary TB are caused by reactivation of a latent TB infection that was acquired earlier in life. It is estimated that one-third of the world population harbors *M. tuberculosis*, which serves as a vast reservoir for propagating this pathogenic organism [14]. The most commonly used tests for diagnosis of latent TB infection are tuberculin test and interferon-γ release assays. However, false-negative and false-positive results with these tests can occur and, therefore, a negative tuberculin test or interferon-γ release assay does not exclude the possibility of TB [15]. In our patient, tests of latent TB infection (tuberculin test and interferon-γ release assay) were both negative despite evidence of active pancreatic TB. Reactivation of latent TB infection can be caused by a variety of factors that weaken the immune system, such as diabetes mellitus, chronic kidney disease, AIDS, immunosuppressive medications and hematologic malignancies. In our patient’s CT images, a calcified lesion was identified in the left lower lung field suggestive of an old healed granuloma. Our patient had a family history of active pulmonary TB (in his mother) which he was exposed to during his childhood. Putting all these findings together, pancreatic TB in our patient was most likely a consequence of reactivation of that latent TB infection, which he acquired from exposure to his mother. No clear immunocompromising factor was identified in our patient. While cases of pancreatic TB have been described in immunocompetent individuals [4], some recent studies suggest that vitamin D deficiency may increase the risk of reactivation in patients with latent TB infection [16]. Our patient had a 25-hydroxy-cholecalciferol level of 8 ng/mL (reference range: 30-80 ng/mL) consistent with severe vitamin D deficiency, which contributed to the reactivation of latent TB infection. Vitamin D deficiency has been reported in a substantial proportion of the South Asian population and a variety of hypotheses have been proposed to explain this observation (although the exact cause remains unknown) [17]. Given that our patient was Nepalese by nationality, we presume that he probably had undiagnosed vitamin D deficiency prior to the acute illness. Although pancreatic insufficiency is known to cause vitamin D deficiency [18], this seems unlikely as the patient did not have any history of diarrhea or steatorrhea, and his duration of illness was relatively short. In some patients with tuberculosis, low levels of 25-hydroxy-cholecalciferol are due to increased 1-α-hydroxylation caused by the production of 1-α-hydroxylase by epithelioid cells in granuloma. However, such patients also tend to have significantly elevated levels of 1,25-dihydroxy-cholecalciferol along with hypercalcemia, which was not the case in our patient. Hypoalbuminemia was also noted in our patient, which is a typical feature of patients with tuberculosis [19].

Pancreatic TB is a readily treatable and curable disease. This is in stark contrast with other causes of a pancreatic mass (such as pancreatic adenocarcinoma) which often have dismal prognoses and are considered incurable [20]. Treatment of pancreatic TB is with first-line anti-tuberculous therapy consisting of a four-drug intensive phase (isoniazid, rifampin, pyrazinamide and ethambutol) for two months followed by a two-drug continuation phase (isoniazid and rifampin) for four months. In the absence of concomitant bone, joint or central nervous system disease, a six-month course of anti-tuberculous therapy is as effective as longer treatment courses. In certain special circumstances (e.g. positive AFB cultures at the end of intensive phase), prolongation and/or modification of the anti-tuberculous regimen may be necessary.

## Conclusions

Pancreatic TB should be considered in the differential diagnosis of a pancreatic mass, especially in patients from areas endemic for TB. FNA of pancreatic lesions using EUS is a valuable tool for obtaining specimens for histopathological and microbiologic evaluation. While pancreatic masses are most commonly malignant in nature, pancreatic TB is unique in that it is readily treatable and curable. As TB is a readily curable disease, distinction from pancreatic cancer is important to avoid unnecessary distress and potentially harmful interventions.
